# Metagenomic and metabolomic analyses show correlations between intestinal microbiome diversity and microbiome metabolites in ob/ob and ApoE^−/−^ mice

**DOI:** 10.3389/fnut.2022.934294

**Published:** 2022-10-13

**Authors:** Sashuang Dong, Chengwei Wu, Wencan He, Ruimin Zhong, Jing Deng, Ye Tao, Furong Zha, Zhenlin Liao, Xiang Fang, Hong Wei

**Affiliations:** ^1^Precision Medicine Institute, The First Affiliated Hospital, Sun Yat-sen University, Guangzhou, China; ^2^College of Food Science, South China Agricultural University, Guangzhou, China; ^3^Guangdong Provincial Key Laboratory of Utilization and Conservation of Food and Medicinal Resources in Northern Region, Shaoguan University, Shaoguan, China; ^4^Shanghai Biozeron Biotechnology Co., Ltd., Shanghai, China

**Keywords:** ob/ob mice, ApoE^−/−^ mice, metagenomic, metabolomic, gut microbe

## Abstract

Obesity and atherosclerosis are the most prevalent metabolic diseases. ApoE^−/−^ and ob/ob mice are widely used as models to study the pathogenesis of these diseases. However, how gut microbes, gut bacteriophages, and metabolites change in these two disease models is unclear. Here, we used wild-type C57BL/6J (Wt) mice as normal controls to analyze the intestinal archaea, bacteria, bacteriophages, and microbial metabolites of ob/ob and ApoE^−/−^ mice through metagenomics and metabolomics. Analysis of the intestinal archaea showed that the abundances of *Methanobrevibacter* and *Halolamina* were significantly increased and decreased, respectively, in the ob/ob group compared with those in the Wt and ApoE^−/−^ groups (*p* < 0.05). Compared with those of the Wt group, the relative abundances of the bacterial genera *Enterorhabdus, Alistipes, Bacteroides, Prevotella, Rikenella, Barnesiella, Porphyromonas, Riemerella*, and *Bifidobacterium* were significantly decreased (*p* < 0.05) in the ob/ob mice, and the relative abundance of *Akkermansia* was significantly decreased in the ApoE^−/−^ group. The relative abundances of *A. muciniphila* and *L. murinus* were significantly decreased and increased, respectively, in the ob/ob and ApoE^−/−^ groups compared with those of the Wt group (*p* < 0.05). *Lactobacillus_ prophage_ Lj965* and *Lactobacillus _ prophage _ Lj771* were significantly more abundant in the ob/ob mice than in the Wt mice. Analysis of the aminoacyl-tRNA biosynthesis metabolic pathway revealed that the enriched compounds of phenylalanine, glutamine, glycine, serine, methionine, valine, alanine, lysine, isoleucine, leucine, threonine, tryptophan, and tyrosine were downregulated in the ApoE^−/−^ mice compared with those of the ob/ob mice. Aminoacyl-tRNA synthetases are considered manifestations of metabolic diseases and are closely associated with obesity, atherosclerosis, and type 2 diabetes. These data offer new insight regarding possible causes of these diseases and provide a foundation for studying the regulation of various food nutrients in metabolic disease models.

## Graphical Abstract

Overall experimental design. 
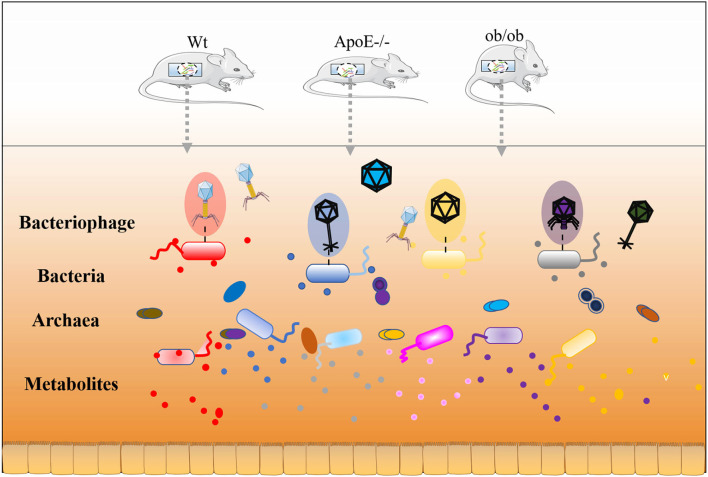

## Introduction

The intestinal microbiota is composed of trillions of bacteria, archaea, and viruses forming complex ecosystems and is regarded as a modulator of host health (1). Bacterial species commonly serve as a commensal ecosystem that benefits host health by facilitating host metabolism, ameliorating immune cells, and providing barrier protection (2). However, ecological disorders can occur if the gut microenvironment is unbalanced (3). Although causality between intestinal dysbacteriosis and metabolic diseases (e.g., obesity and atherosclerosis) has been extensively reported (4), the mechanism of the pathogenesis remains unclear. Accumulating studies have shown that intestinal dysbacteriosis is associated with key tripartite interaction between bacteriophages and their bacterial and human hosts (5).

Atherosclerosis, a chronic inflammatory disease thought to result from intestinal flora disorders, is becoming prevalent globally (6). The relative abundances of Enterobacteriaceae, *Streptococcus, Clostridium*, and other microorganisms are significantly increased in the intestinal tracts of patients with coronary atherosclerosis, thus inhibiting enrichment of beneficial intestinal bacteria. Additionally, the abundances of *Streptococcus* and Enterobacteriaceae are positively correlated with blood pressure and myocardial indices, respectively (7, 8). Although the exact pathogenesis of atherosclerosis is complex and unclear, inflammation, especially the release of proinflammatory cytokines by macrophages infiltrated by atherosclerotic plaques, is believed to be pivotal (4). ApoE^−/−^ and ob/ob mice are often used as animal models to study the pathogenesis of metabolic diseases (7). Epidemiological studies have shown that the microbial compositions of ApoE^−/−^and ob/ob mice differ significantly from those of normal controls and are characterized by low abundances of butyrate-producing bacteria and increased opportunistic pathogens (4, 9). Leptin is the main metabolite of adipocytes, which regulates energy homeostasis, bone growth, and immune responses. Moreover, leptin-impaired signal transduction is closely related to metabolic diseases, including obesity and type 2 diabetes (10). Researchers have used ob/ob mice as metabolic disease models to study how probiotics regulate the intestinal flora and determine whether improvements in the intestinal flora are related to amelioration of lipid and glucose metabolism (9, 11).

ApoE^−/−^ and ob/ob mice are commonly used as model organisms to clarify the roles of dietary practices in obesity and atherosclerosis treatment. Several studies revealed that dietary intake can relieve these metabolic diseases by modulating the composition and structure of the host's gut microbiota (12–14). However, changes in intestinal bacteria, bacteriophages, and metabolomics of mice with metabolic diseases remain unclear (15, 16). Here, we used metagenomic and metabolomic methods to study the composition and structures of intestinal bacteria and phages and the intestinal metabolomic changes in ob/ob and ApoE^−/−^ mice compared with those of wild-type (Wt) mice. Our results provide insight for further studying the pathophysiology and pharmacology of metabolic diseases such as obesity and atherosclerosis.

## Materials and methods

### Experimental design

Thirty-six-week-old homozygous male mice were purchased from GemPharmatech Co., Ltd. (Jiangsu, China): ten *ApoE*-deficient mice (B6/JGpt-Apoeem1Cd82/Gpt; ApoE^−/−^ group), ten obese leptin-deficient mice (B6/JGpt-Lepem1Cd25/Gpt; ob/ob group), and ten Wt mice (C57BL/6JGpt; Wt group). Body weight and blood glucose levels differed significantly among the three groups (*p* < 0.05; Supplementary Figure S1). The normal animal diet (D12450J) was prepared by Jiangsu Xietong Pharmaceutical Bioengineering Co., Ltd. (Supplementary Table S1). All animal experiments were performed in the Animal Center of South China Agricultural University. Mice were fed the D12450J diet for 1 week of dietary acclimation, before exposure to a 7-day D12450J feeding. Three or four mice were grouped and fed in a cage with poplar bedding under controlled conditions (temperature: 23 ± 2°C, humidity: 70–75%, and a 12-h/12-h light-dark cycle). Poplar bedding and drinking water were refreshed every 2 days. Water/food consumption and changes in body weight were monitored three times per week. Animal experiments were conducted under National Institute of Health (NIH) guidelines (NIH Publication No. 85-23 Rev. 1985) under supervision of the Animal Experimentation Ethics Review Committee of South China Agricultural University (Guangzhou, China).

### Sample collection, DNA extraction, and sequencing

Fecal samples were collected from all mice 2 weeks after feeding and immediately frozen at −80°C for experimentation. DNA was extracted from the fecal samples using the E.Z.N.A.^®^ stool DNA kit (Omega Bio-tek, Norcross, GA, USA) per the manufacturer's protocols. Briefly, DNA buffer was added to the sample for viral capsid lysis and purified through spin-column. The extracted DNA was eluted with TE buffer. The DNA concentration and purity were determined using a Nanodrop 2000 (Thermo Scientific, USA) and stored at −80°C until sequencing. Metagenomic shotgun sequencing libraries were constructed and sequenced at Shanghai Biozeron Biological Technology Co., Ltd. The sequencing libraries were constructed using a Nextera XT DNA Library Preparation kit (Illumina). High-sensitivity double-stranded DNA kits were used to determine the concentrations of all libraries on a Qubit Fluorometer (Thermo Fisher Scientific). After sequencing in the Illumina NovoSeq instrument in pair-end 150-bp (PE150) mode, quality control was performed using Trimmomatic (http://www.usadellab.org/cms/uploads/supplementary/Trimmomatic) on raw sequence reads to remove adaptor contaminants and low-quality reads. Using the BWA mem algorithm with M-k 32 -t 16 parameters (http://bio-bwa.sourceforge.net/bwa.shtml), quality-control reads were mapped to the mouse genome (NCBI). After removing host-genome contaminants and low-quality data, the clean reads were used for further analysis.

### Reads-bases phylogenetic annotation

According to the default database downloaded from Broad Institute (min-score-identity = 0.90, identity margin = 0.02), taxonomy of the clean reads for each sample was measured through the PathSeq pipeline distributed in GATK v4.1.3 (https://github.com/usadellab/Trimmomatic) (17). By default, alignments were discarded in PathSeq once two read pairs did not point to the same organism. All bacteriophage, archaeal, and bacterial genome sequences in the NCBI RefSeq database were consistent with those in the taxonomy database. All reads were then classified into seven phylogenetic levels: domain, phylum, class, order, family, genus, and species or unclassified. Annotations generated in PathSeq were used to construct the host-genome and phage relationships.

### Alpha- and beta-diversity analyses

To determine the diversity indices, including the richness and Shannon diversity indices, rarefaction analysis was performed using Mothur v.1.21.1. Beta-diversity analysis was conducted through the community ecology package, *vegan*, in R. Bray-Curtis distance matrices with 999 permutations was applied to measure the virome community similarity using *vegan* in R. Based on a Spearman's rank correlation coefficient >0.6 and *p* < 0.05, correlations between the virus and other elements (other species and metabolites) were determined in R. The relationships were visualized using a correlation heatmap and network diagrams constructed in Gephi (https://gephi.org).

### Metabolomic profiling analysis

Targeted metabolomic analysis of the fecal samples was performed using Metabo-Profile (Shanghai, China). The metabolites were detected according to previously published references (18). The sample preparation procedures were performed as per previously published methods with minor modifications (19). UPLC-MS/MS (ACQUITY UPLC-Xevo TQ-S, USA) was used to quantify the microbial metabolites (20). Reserve solutions of all 164 representative reference chemicals of the microbial differential metabolites were prepared in methanol, ultrapure water, or sodium hydroxide solution as per the internal standards (Supplementary Table S2). Internal standards were added to the samples to monitor data quality and compensate for matrix effects (21). After generating raw data files from the UPLC-MS/MS, peak integration, calibration, and quantitation were performed for each metabolite using MassLynx software (v4.1, Waters, Milford, MA, USA) (22). The iMAP platform (v1.0; Metabo-Profile, Shanghai, China) was used for statistical analysis. Principal component analysis (PCoA) and orthogonal partial least square discriminant analysis (OPLS-DA) (23) were used to visualize the metabolic differences among the experimental groups. The biological patterns, functions, and pathways of the differentially expressed metabolites were analyzed using the Matabo Analyst online tool (version 4.0) (24).

### Fecal metabolomic analysis

Thawed fecal samples (5 mg) were dispersed in 25 μL of water and homogenate with zirconium oxide beads for 3 min, before metabolite extraction with 120 μL of a mixture of methanol and internal standard. The homogenate process was repeated once, then the mixture was centrifuged at 18000 × *g* for 20 min. Next, 20 μL of supernatant was transferred to a 96-well plate. The subsequent procedures were performed on an Eppendorf epMotion Workstation (Eppendorf Inc., Humburg, Germany). The plate was sealed, and derivatization was performed at 30°C for 60 min. Next, 330 μL of ice-cold 50% methanol solution was added to dilute the sample. The plate was then stored at −20°C for 20 min, then centrifuged at 4000 × *g* at 4°C for 30 min. The supernatant (135 μL) was then transferred to a new 96-well plate with 10 μL of internal standards in each well. The derivatized stock standards were then serially diluted.

### Correlation analysis

**Figure 1:**
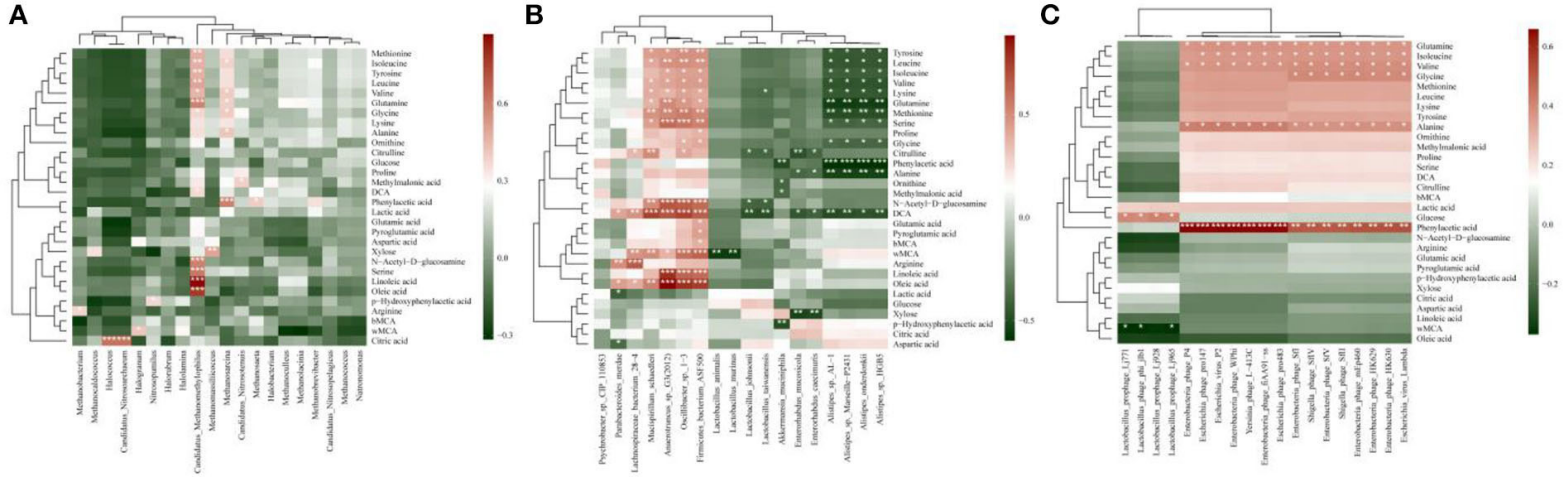
Correlation analysis of differential metabolites and intestinal microorganisms. Correlation analyses of differential metabolites with abundances >1% and archaea **(A)**, bacteria **(B)**, and bacteriophages **(C)** with relative abundances >1%. The correlation (*r*-value) was calculated and is shown in red to green, representing positive and negative correlations, respectively.

Spearman's rank correlations and their significances were calculated using the cor and cor.test functions in R, respectively (25). The correlation (*r*-value) was calculated and is shown in yellow to blue, representing positive and negative correlations, respectively (Figure 1).

### Statistical analysis

GraphPad Prism 8.3 (GraphPad Software, La Jolla, CA, USA) and TBtools software (26) were used to construct the graphs. All data are expressed as means ± standard deviation. A two-tailed Wilcoxon's rank-sum test was used to identify statistically significant differences between two groups using SPSS (version 23.0, Chicago, IL, USA). *P* < 0.05 was considered statistically significant.

## Results

### Overall intestinal microbiota diversity in the ApoE^–/-^, ob/ob, and Wt Mice

**Figure 2:**
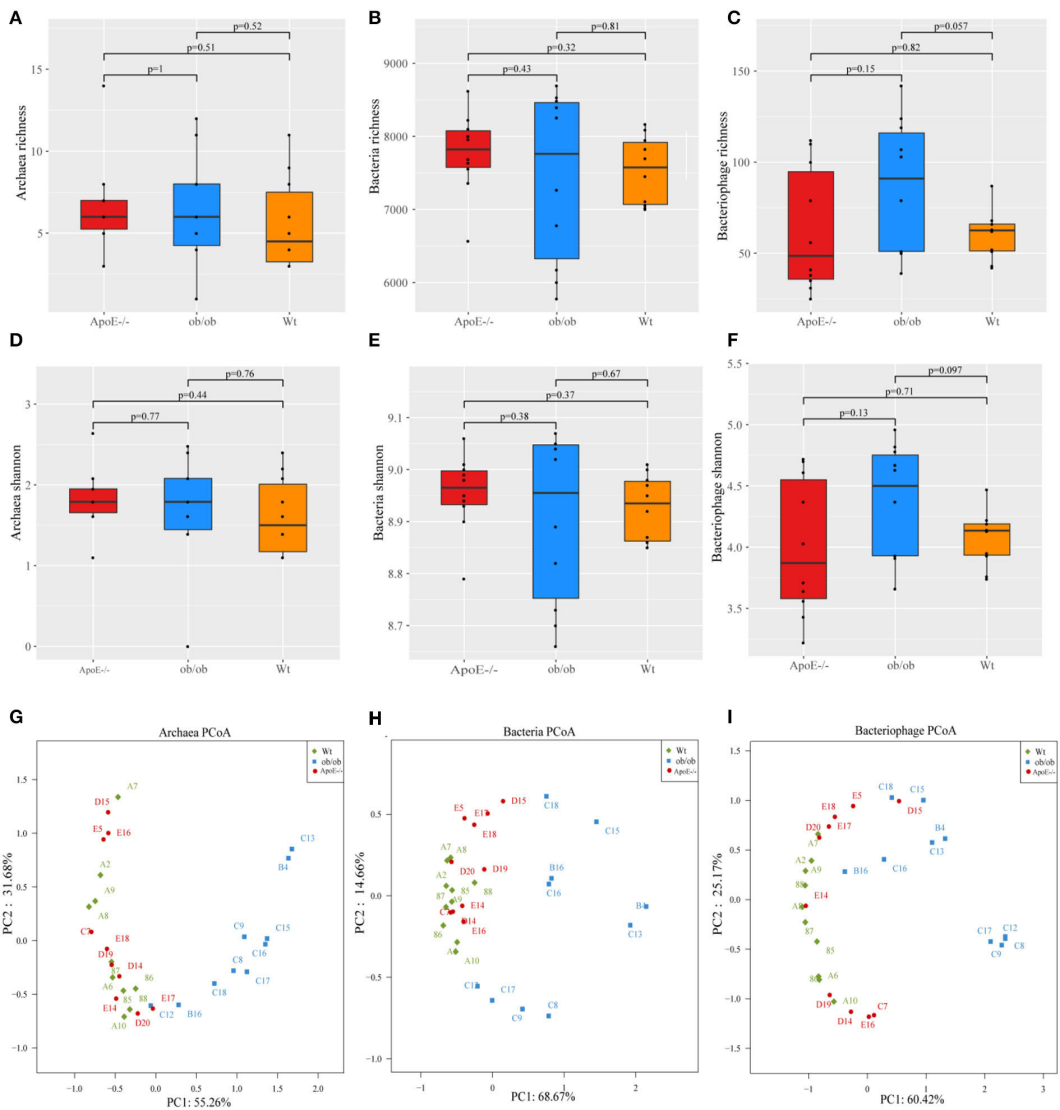
Microbiome alpha- and beta-diversity indices. **(A–C)** Archaeal, bacterial, and bacteriophage richness; **(D–F)** Shannon indexes for archaea, bacteria, and bacteriophages; **(G–I)** Scatter plot of principal component (PC) analysis. PC1 and PC2 clusterings for each group.

The intestinal microbiotas composed of archaea, bacteria, and bacteriophages were characterized by metagenomic sequencing. Alpha diversity (e.g., richness and Shannon indices) was used to characterize variations in the gut microbiotas. Archaeal, bacterial, and bacteriophage diversity did not differ significantly between the ApoE^−/−^, ob/ob, and Wt mice (*p* > 0.05; Figures 2A–F).

Next, we further analyzed the differences in species distributions among the three mouse groups. Figures 2G–I show the differences in species abundance compositions among groups. The β-diversity data are shown through PCoA plot of the weighted UniFrac distance. Analysis of the intestinal microbial archaea, bacteria, and bacteriophages of the three groups showed that as the sample points became closer on the coordinate axis, the species abundance compositions among the samples became more similar in the corresponding dimension. Archaeal principal components 1 and 2 explained 55.26 and 31.68% of the changes, respectively (Figure 2G). Bacterial principal components 1 and 2 explained 68.67 and 14.66% of the changes, respectively (Figure 2H). Bacteriophage principal components 1 and 2 explained 60.42 and 25.17% of the changes, respectively (Figure 2I).

Overall, these data have shown that the diversity of Archaeal, bacterial, and bacteriophage were no significant difference, but there were differences in species composition in the ApoE^−/−^, ob/ob, and Wt mice, indicating that ApoE-deficient and obese leptin-deficient have certain effects on the composition of gut microbes.

### Gut archaeal compositions in the ApoE^–/-^, ob/ob, and Wt mice

**Figure 3:**
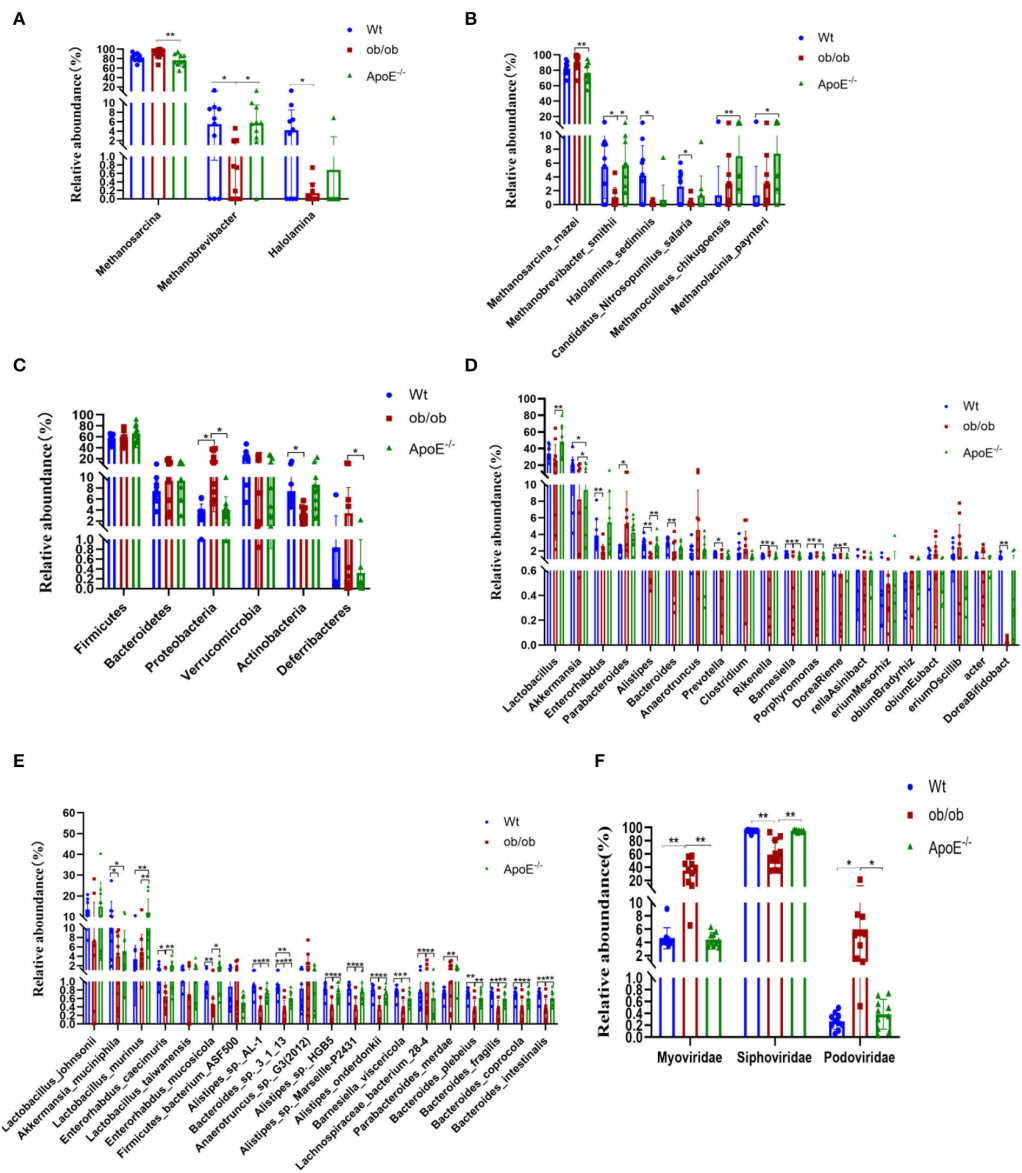
Relative abundances of archaea, bacteria and bacteriophages at the phylum, family, genus and species levels and their significantly different compositions in Wt, ob/ob, and ApoE^−/−^ mice. **(A,B)** Relative abundances of archaea at the genus and species levels. **(C)** Relative abundances of bacteria at the phylum level. **(D,E)** Relative abundances of bacteria at the genus and species levels. **(F)** Relative abundances of bacteriophages at the family level.

Thirty stool samples were shotgun sequenced through the Illumina MiSeq platform and analyzed using metagenomics (Figure 3). Intestinal archaea were mainly composed of the phyla Euryarchaeota and Thaumarchaeota (Supplementary Figure S2A) in all three groups. The relative Euryarchaeota abundances were 97.40 ± 2.36%, 99.66 ± 0.69% and 98.16 ± 3.34% for the Wt, ob/ob and ApoE^−/−^ groups, respectively. Supplementary Figures S2B,C shows the relative abundances of the top six gut archaea at the genus and species levels. *Methanosarcina, Methanobrevibacter*, and *Halolamina* were the dominant intestinal archaeal genera in the Wt, ob/ob, and ApoE^−/−^ groups, with relative abundances of 82.19 ± 8.30%, 90.77 ± 10.27%, and 76.47 ± 12.51%; 5.46 ± 4.55%, 1.01 ± 1.47, and 5.71 ± 3.82%; and 4.18 ± 4.33%, 0.13 ± 0.24%, and 0.68 ± 2.16%, respectively (Figure 3A). The relative abundance of *Methanosarcina* was significantly increased in the ob/ob group compared with that of the ApoE^−/−^ group (*p* < 0.05). The relative abundance of *Methanobrevibacter* was significantly decreased in the ob/ob group compared with that of the Wt and ApoE^−/−^ groups (*p* < 0.05), and the relative abundance of *Halolamina* was significantly decreased in the ob/ob group compared with that of the Wt group (Figure 3A). Figure 3B shows the intestinal archaeal species compositions with relative abundances of >1%. Five species belonged to Euryarchaeota, of which, *Methanobrevibacter_smithii* and *Halolamina_sediminis* were significantly decreased in the ob/ob group compared with those of the Wt group; *Methanoculleus_chikugoensis* and *Methanolacinia_paynteri* were significantly increased in the ApoE^−/−^ group compared with those of the Wt group, and *Methanosarcina_mazei* was significantly decreased in the ob/ob group compared with that of the ApoE^−/−^ group (Figure 3B). One species from Thaumarchaeota, *Candidatus_Nitrosopumilus_salaria*, was significantly decreased in the ob/ob group compared with that of the Wt group (Figure 3B).

Overall, compared with that of the ApoE^−/−^ group, the relative abundance of Methanosarcina was significantly increased in the ob/ob group, which indicated that this Methanosarcina is related to obese leptin-deficient.

### Gut bacterial compositions in the ApoE^–/-^, Ob/ob, and Wt mice

Supplementary Figures S3A–C show the intestinal bacterial compositions at the phylum, genus, and species levels to illustrate the specific changes in microbial communities in the ApoE^−/−^ and ob/ob mice compared with those of the Wt mice. At the phylum level, the relative abundances of Firmicutes, Bacteroidetes, and Verrucomicrobia did not significantly differ among the groups (*p* > 0.05; Figure 3C). The relative abundance of Proteobacteria was significantly increased in the ob/ob group compared with that in the ApoE^−/−^ and Wt groups (*p* < 0.05). The relative abundance of Actinobacteria was significantly decreased in the ob/ob group compared with that of the Wt group, and the relative abundance of Deferribacteres in the ob/ob mice was significantly decreased compared with that of the ApoE^−/−^ group (*p* < 0.05). Figure 3D shows the top 20 dominant bacterial genera (relative abundance >1.00%). At the genus level, the relative abundances of *Lactobacillus* and *Akkermansia* were predominant. Compared with the Wt group, the relative abundances of *Enterorhabdus, Alistipes, Bacteroides, Prevotella, Rikenella, Barnesiella, Porphyromonas, Riemerella*, and *Bifidobacterium* were significantly decreased (*p* < 0.05) in the ob/ob group, and the relative abundance of *Akkermansia* was significantly decreased in the ApoE^−/−^ group. Compared with the ob/ob group, the relative abundances of *Lactobacillus, Akkermansia, Alistipes, Rikenella, Barnesiella, Porphyromonas*, and *Riemerella* were significantly higher in the ApoE^−/−^ group (Figure 3D). Figure 3E shows the compositions of the top 20 species, including six species from Firmicutes, 11 from Bacteroidetes, two from Actinobacteria, and one from Verrucomicrobia. *Lactobacillus_murinus* (*L. murinus*) and *Akkermansia_muciniphila* (*A. muciniphila*) had the highest relative abundances in the three groups (Figure 3E). Compared with the Wt group, the relative abundances of *A. muciniphila* and *L. murinus* were significantly decreased and increased (*p* < 0.05) in the ob/ob and ApoE^−/−^ groups, respectively. These species may be the key species of metabolic diseases (Figure 3E).

Overall, these data have shown that the gut dominant microbiota have changed after knockout of ApoE-deficient and obese leptin-deficient, which may be closely associated with metabolic diseases.

### Gut bacteriophage composition and associations between phages and their bacterial host in ApoE^–/-^, ob/ob, and Wt mice

Supplementary Figures S4A,B shows the differences in intestinal bacteriophage community structures among the three mouse groups. At the family level, the most common bacteriophages were Siphoviridae (94.85 ± 2.08%, 58.85 ± 21.92% and 4.43 ± 1.06%), Myoviridae (4.62 ± 1.59%, 34.44 ± 17.63% and 94.90 ± 1.33%), and Podoviridae (0.27 ± 0.14%, 5.98 ± 6.02% and 0.39 ± 0.25%) in the Wt, ob/ob, and ApoE^−/−^ groups, respectively (Figure 3F). Compared with the Wt group, the relative abundances of Myoviridae and Podoviridae were significantly increased, and the relative abundance of Siphoviridae was significantly decreased in the ob/ob and ApoE^−/−^ mice (Figure 3F). Compared with the ob/ob group, the relative abundances of Myoviridae and Podoviridae were significantly increased, and the relative abundance of Siphoviridae was significantly decreased in the ApoE^−/−^ mice (Figure 3F).

**Figure 4:**
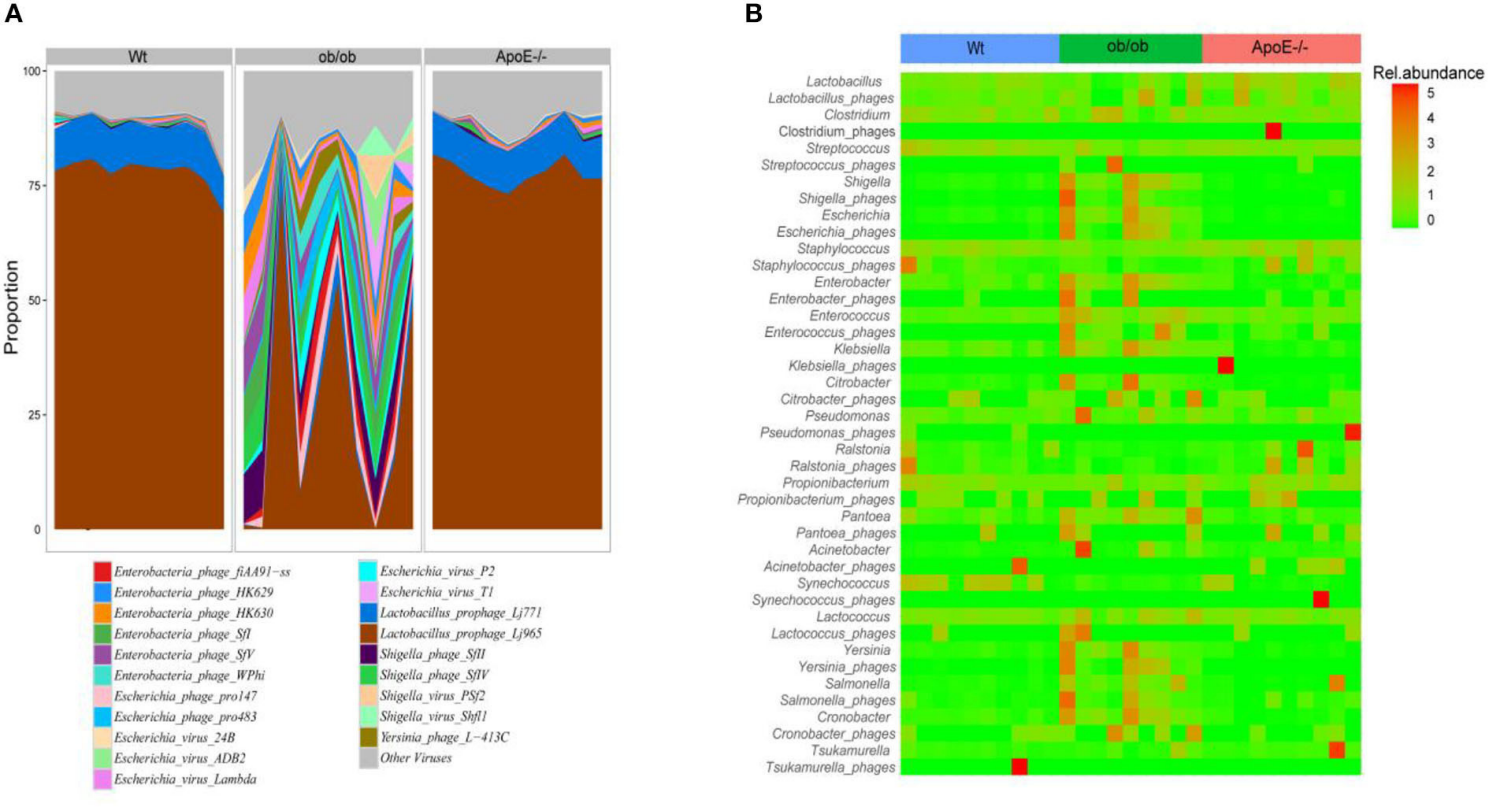
Proportions of bacteriophage species and matched alignments with their bacterial hosts and association predictions between the bacteriophages and bacteria. **(A)** The main bacteriophages were classified according to annotation results. The average relative abundances of these aggregated bacteriophages were calculated in phage reads with known bacterial hosts. **(B)** Abundance heatmap of bacteriophages and their host bacteria, showing the relative abundances of the 21 most abundant bacteriophages in the three mouse groups.

We summarized the bacteriophage species according to their known bacterial hosts. The relative abundances of the predominant bacteriophage species *Lactobacillus_ prophage_ Lj965* and *Lactobacillus _ prophage _ Lj771* in the Wt and ApoE^−/−^ mice were significantly higher than those in the ob/ob mice. *Escherichia_virus_Lambda, Enterobacteria_phage_HK630, Enterobacteria_ phage_HK629, Escherichia_virus_24B, Shigella_phage_SfII, Shigella_phage_SfIV, Enterobacteria_phage_SfV, Enterobacteria_phage_SfI, Escherichia_phage_pro147, Enterobacteria_phage_fiAA91-ss, Escherichia_virus_P2, Escherichia_phage_pro483, Yersinia_phage_L-413C, Enterobacteria_phage_WPhi, Escherichia_virus_ADB2, Escherichia_virus_T1*, and *Shigella_virus_ PS* were the predominant intestinal phages in the ob/ob mice (relative abundance >1%). These species had relative abundances of < 0.3 and 0.6% in the Wt and ApoE^−/−^ mice, respectively (Figure 4A).

The heatmap of the bacteriophages and their host bacteria shows the relative abundances of the 21 most abundant bacteriophages in the three groups (Figure 4B). The bacteriophage/bacteria relationship was characterized by high abundances of some phages and decreased bacterial host abundances in others. Compared with those of the Wt mice, *Shigella_phages, Enterobacter_phages, Enterococcus_phages*, and *Klebsiella_phages* had lower abundances, and *Shigella, Enterobacter, Enterococcus*, and *Klebsiella* had higher abundances in the ob/ob mice (Figure 4B). *Ralstonia_phages* and *Propionibacterium_phages* were highly abundant, but the corresponding *Ralstonia* and *Propionibacterium* bacteria had low abundances in the ApoE^−/−^ mice (Figure 4B). *Escherichia* and *Escherichia_phages* had less stringent relationships in only a few ob/ob mice, and *Staphylococcus* and *Staphylococcus_phages* had less stringent relationships in only a few ApoE^−/−^ mice (Figure 4B).

Our study showed that the relative abundance of intestinal phage Podoviridae, *Lactobacillus*_*prophage_Lj965*, and *Lactobacillus_prophage_Lj771* were significantly elevated in ob mice, which are closely associated with metabolic diseases, such as ulcerative colitis and type 2 diabetes.

### Compositions and differential analyses of targeted fecal metabolites in Wt, ob/ob, and ApoE^–/-^ mice

**Figure 5:**
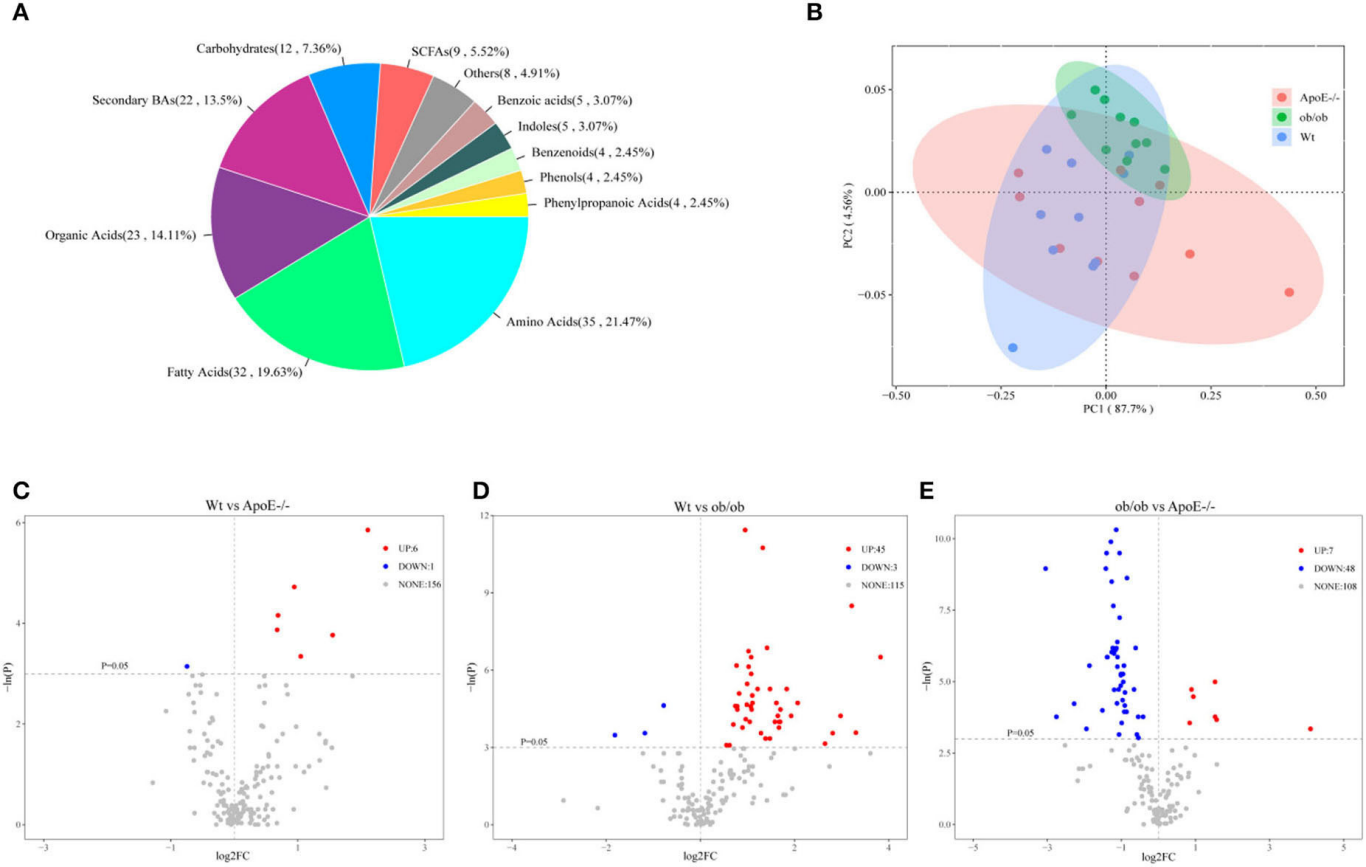
Targeted fecal metabolomics analysis of the Wt, ApoE^−/−^, and ob/ob mice. **(A)** Metabolite divisions among the three mouse groups. **(B)** Scatter plot of the principal component (PC) analysis. PC1 and PC2 clusterings for each group. **(C–E)** Volcano plot of differentially expressed genes for Wt vs ApoE^−/−^**(C)**, Wt vs ob/ob **(D)**, and ob/ob vs ApoE^−/−^**(E)**, with *p* < 0.05 and log2 fold-change >0. Each point represents a metabolite. Red and blue dots indicate upregulation and downregulation, respectively; gray dots indicate no statistical difference.

Targeted UPLC-MS/MS analyses of the feces from Wt, ob/ob, and ApoE^−/−^ mice revealed 163 metabolites, mainly including carbohydrates (7.36%), amino acids (21.47%), secondary bases (13.5%), organic acids (14.11%), fatty acids (19.63%), short-chain fatty acids (5.52%), phenols (2.45%), benzenoids (2.45%), benzoic acids (3.07%), phenylpropanoic acids (2.45%), and indoles (3.07%; Figure 5A). Supplementary Figure S5 shows the relative abundance of each metabolite class in each group. The abundance patterns of the metabolites differed significantly among the groups by PCoA analysis (Figure 5B). Compared with those of the Wt mice, acetoacetic acid, 3-hydroxybutyric acid, xylulose, ribulose, tartaric acid, and 3-hydroxyphenylacetic acid-3 were significantly upregulated in the ApoE^−/−^ mice (Figure 5C), and deoxycholic acid (DCA), lithocholic acid (LCA), glycodeoxycholic acid (GDCA), glutamine, α-ketoisovaleric acid, and butyric acid were significantly upregulated in the ob/ob mice (Figure 5D). Compared with those of the ob/ob mice, lysine, citrulline, DCA, eicosapentaenoic acid (EPA), GDCA and glutamine were downregulated in the ApoE^−/−^ mice (Figure 5E).

Overall, these data have shown that the key metabolites in intestinal microorganisms of ApoE-deficient and obese leptin-deficient mice with metabolic diseases may play causal roles in the pathophysiology of metabolic diseases.

### Metabolites potential biomarkers and metabolic pathway analysis

**Figure 6:**
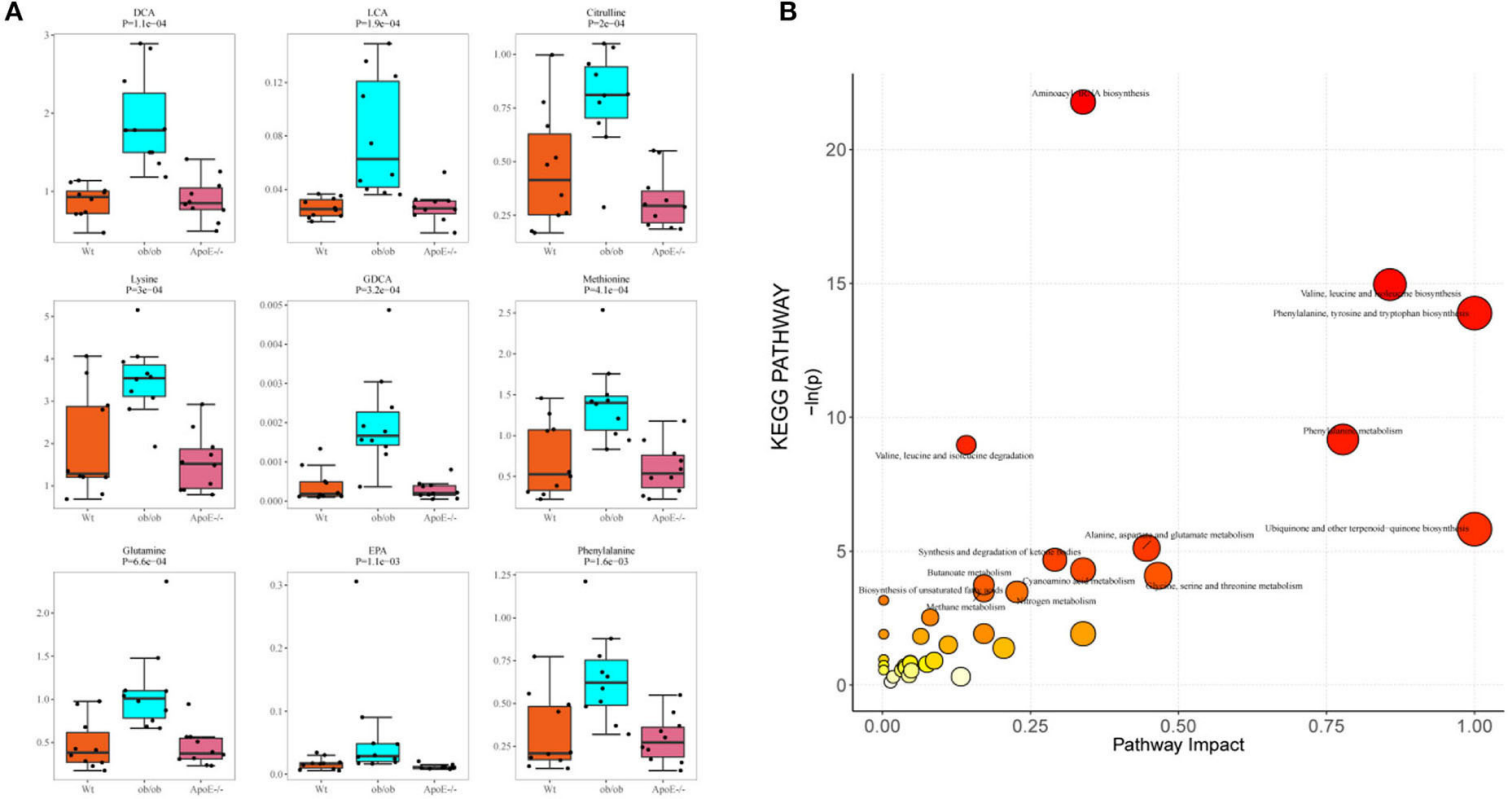
Potential biomarker metabolites and KEGG pathway analysis. **(A)** Box-scatter plot of potential biomarker metabolites of the top 9 differential metabolites ordered by *p*-value; **(B)** Bubble plot of enriched KEGG pathways. Bubble size represents the number of metabolites enriched in the pathway.

Figure 6A shows the top nine differential potential biomarker metabolites (*p* < 0.05). The common metabolites among the Wt, ApoE^−/−^, and ob/ob mice were DCA, LCA, lysine, citrulline, EPA, GDCA, glutamine, methionine, and phenylalanine.

A pathway-associated metabolite sets library pathway enrichment analysis was conducted for the three groups. The metabolite pathway analysis suggested that these pathways were mainly involved in aminoacyl-tRNA biosynthesis; valine, leucine, and isoleucine biosynthesis; phenylalanine, tyrosine, and tryptophan biosynthesis; phenylalanine metabolism; valine, leucine, and isoleucine degradation; ubiquinone and other terpenoid-quinone biosynthesis; alanine, aspartate, and glutamate metabolism; synthesis and degradation of ketone bodies; cyanoamino acid metabolism; glycine, serine, and threonine metabolism; butanoate metabolism and biosynthesis of unsaturated fatty acids (Figure 6B, Supplementary Figure S6). Differential expressions in the synthesis and degradation of ketone bodies pathways in the Wt and ApoE^−/−^ mice showed that acetoacetic acid was significantly upregulated in the ApoE^−/−^ mice (Supplementary Figure S7A). The Kyoto Encyclopedia of Genes and Genomes (KEGG) metabolites pathway of aminoacyl-tRNA biosynthesis was differentially regulated in the Wt and ob/ob mice (Supplementary Figure S7B). Asparagine, histidine, phenylalanine, glutamine, serine, methionine, valine, alanine, lysine, leucine, threonine, and tyrosine were significantly upregulated, and aspartic acid was downregulated in the ob/ob mice. Comparing the aminoacyl-tRNA biosynthesis metabolic pathways showed that the enriched compounds of phenylalanine, glutamine, glycine, serine, methionine, valine, alanine, lysine, isoleucine, leucine, threonine, tryptophan, and tyrosine were downregulated in ApoE^−/−^ mice compared with those of the ob/ob mice (Supplementary Figure S7C).

### Microbiota correlation analysis of the Wt, ob/ob, and ApoE^–/-^ mice

We performed correlation analyses of the archaebacteria, bacteria and bacteriophages, and metabolites. *Candidatus_Nitrosoarchaeum* and *Halococcus* were significantly positively correlated with citric acid (*p* < 0.05), and *Candidatus_Methanomethylophilus* was significantly positively correlated with oleic acid, linoleic acid, and serine (*p* < 0.05; Figure 1A). *Lachnospiraceae_bacterium_28-4* was significantly correlated with arginine (*p* < 0.05). *Anaerotruncus_sp._G3(2012)* was significantly positively correlated with DCA, serine, oleic acid, linoleic acid, and N-acetyl-D glucosamine. *Firmicutes_bacterium_ASF500* was significantly positively correlated with α-muricholic acid (α-MCA), DCA, serine, oleic acid, linoleic acid, and N-acetyl-D-glucosamine. *Lachnospiraceae_bacterium_28-4* was significantly positively correlated with arginine. *Mucispirillum_schaedleri* was significantly positively correlated with DCA and oleic acid. *Oscillibacter_sp._1-3* was significantly positively correlated with oleic acid, linoleic acid, N-acetyl-D-glucosamine, wMCA, DCA, serine, and methionine (*p* < 0.05).

Significant negative correlations were found between *Lactobacillus_animalis* and wMCA; *Lactobacillus_murinus* and wMCA; *Enterorhabdus_mucosicola* and xylose; *Alistipes_sp._AL-1* and phenylacetic acid, glutamine and alanine; *Alistipes_sp._MarseilleP2431* and phenylacetic acid, glutamine and alanine; *Alistipes_onderdonkii* and phenylacetic acid, alanine and glutamine; and *Alistipes_sp._HGB5* and glutamine, alanine and phenylacetic acid (*p* < 0.05; Figure 1B). *Enterobacteria_phage_fiAA91-ss, Enterobacteria_phage_P4, Enterobacteria_phage_WPhi, Escherichia_phage_pro147, Escherichia_phage_pro483, Escherichia_virus_P2*, and *Yersinia_phage_L-413C* were significantly positively correlated with phenylacetic acid (Figure 1C).

### Correlations between intestinal microbes and metabolites in Wt, ob/ob, and ApoE^–/-^mice

**Figure 7:**
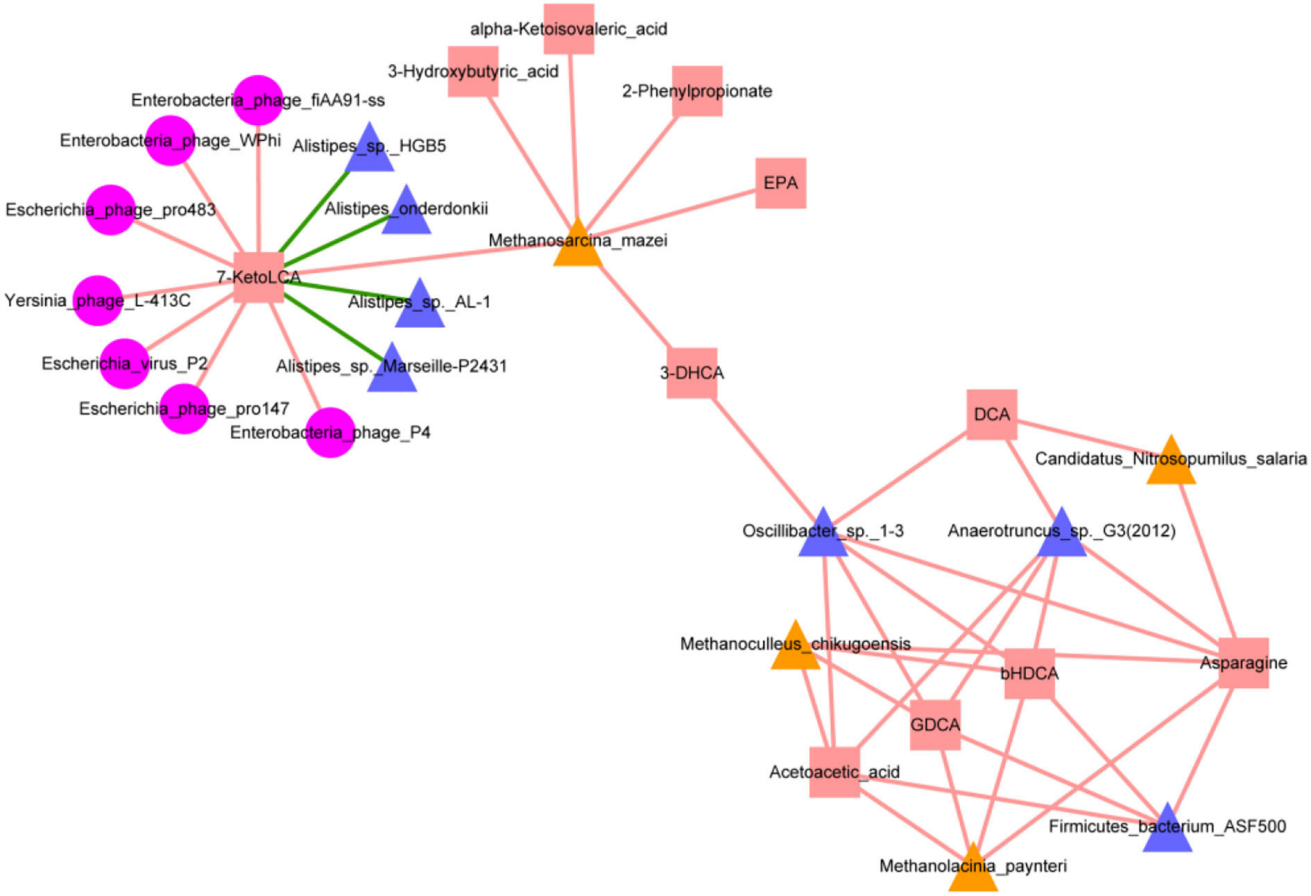
Interaction network of metagenomic and metabolomic features for mice with metabolic diseases (ApoE^−/−^ and ob/ob) groups and wild-type (Wt) control group. Red squares represent metabolites; yellow triangles represent archaea; pink circles represent bacteriophages; blue-purple triangles represent bacteria. Red and green lines represent positive and negative correlations, respectively. Interactions were visualized using Cytoscape.

To further examine extended network links, we evaluated archaeal, bacterial, and bacteriophage abundances for associations with key metabolite levels. The resulting network contained 30 nodes and 43 edges, representing significant correlations among archaea, bacteria, bacteriophages, and metabolites (Figure 7). *Alistipes_onderdonkii, Alistipes_sp._AL-1, Alistipes_sp._HGB5*, and *Alistipes_sp._Marseille-P2431* were significantly negatively correlated with 7-KetoLCA (*p* < 0.05). Significant positive correlations were found between *Anaerotruncus_sp._G3(2012)* and DCA, asparagine and GDCA; *Firmicutes_bacterium_ASF500* and asparagine, acetoacetic acid, bHDCA and GDCA; and *Oscillibacter_sp._1-3* and 3-DHCA, asparagine, DCA, acetoacetic acid, bHDCA, and GDCA; *Candidatus_Nitrosopumilus_salaria* and asparagine and DCA; *Methanoculleus_chikugoensis* and acetoacetic acid and bHDCA; *Methanolacinia_paynteri* and acetoacetic acid, bHDCA, asparagine, and GDCA; *Methanosarcina_mazei* and 7KetoLCA; and *Methanoculleus_chikugoensis* and asparagine and GDCA (*p* < 0.05). *Enterobacteria_phage_fiAA91ss, Enterobacteria_phage_P4, Enterobacteria_phage_WPhi, Escherichia_phage_pro147, Escherichia_phage_pro483, Escherichia_virus_P2*, and *Yersinia_phage_L-413C* were significantly positively correlated with 7-KetoLCA (*p* < 0.05).

In summary, our multi-omics analysis provides fundamental data for investigating the causal relationship of key microbial species and their metabolites in the occurrence and development of metabolic diseases, especially obesity. Metabolite pathway analysis showed that these metabolite pathways were mainly involved in the biosynthesis of aminoacyl-tRNA and were widely present in organisms. These data offer new insights regarding possible causes of these diseases and provide a foundation for studying the regulation of various food nutrients in metabolic disease models.

## Discussion

ApoE^−/−^ and ob/ob mice are widely used to study the pharmacology and pathogeneses of metabolic diseases (4). Here, we measured the weight and blood glucose levels of the three mouse groups and found significant differences in ob/ob and ApoE^−/−^ mice compared with those of Wt mice (Supplementary Figure S1), indicating that gene knockout strongly affects the weight and blood glucose levels of mice. We described the structure and compositions of the gut archaea, bacteria, bacteriophages, and metabolites in the ob/ob and ApoE^−/−^ mice compared with those of the Wt mice. To our knowledge, this is the first study to focus on the gut archaea, bacteria, bacteriophages, and metabolites of ob/ob and ApoE^−/−^ mice. These data enable better understanding obesity, cardiovascular disease, diabetes, and their related treatments.

Our results showed no significant differences in microbial diversity among ob/ob and ApoE^−/−^ mice compared with those of Wt mice (*p* > 0.05), likely because the ob/ob and ApoE^−/−^ mice were still young and showed early subtle pathological symptoms (Figures 2A–F). The relative abundance of *Methanosarcina* was significantly increased in the ob/ob group compared with that of the ApoE^−/−^ group (*p* < 0.05). Studies have increasingly focused on the metabolism of *Methanosarcina* fermentation products (methane) in the intestines, mainly focusing on the relationship between *Methanosarcina* and intestinal dysfunction. The number of intestinal methanogens in patients with irritable bowel syndrome is often less than that of the normal population. Intestinal methanogens have also been associated with obesity (27).

Compared with the Wt group, the relative abundances of *A. muciniphila* and *L. murinus* were significantly decreased and increased (*p* < 0.05) in the ob/ob and ApoE^−/−^ groups, respectively. These may be key species in metabolic diseases (Figure 3E). *A. muciniphila* is a typical species of intestinal bacteria. *Akkermansia* belongs to the Verrucomicrobia phylum (28) and human intestinal mucin-degrading bacteria (29). Decreased abundances of intestinal *Akkermansia* in metabolic diseases may be related to diet-induced obesity, type 2 diabetes, liver injury, and other metabolic disorders (23, 30). Previous studies have shown decreased abundances of *A. muciniphila* in the guts of patients with ulcerative colitis and metabolic disorders, suggesting that *A. muciniphila* may have potential anti-inflammatory properties (31). When ApoE^−/−^ mice were treated with *A. muciniphila* for 8 weeks after consuming a western-type diet, their lipid metabolism did not change, but the expressions of proinflammatory cytokines and intercellular adhesion molecule 1 (ICAM-1) in the aorta decreased, and infiltration of macrophages into aortic atherosclerosis decreased (7, 32). These results suggest that *A. muciniphila* can positively regulate the intestinal microflora (33), improve intestinal barrier functions, and protect against obesity and atherosclerosis (34). However, further research is needed to explore the correlation between *A. muciniphila* and ulcerative colitis and metabolic diseases, especially in humans. Studies have demonstrated that *L. murinus* can reduce inflammation associated with aging in calorie-restricted mice (35), and its abundance is significantly decreased in the intestinal tracts of cirrhotic rats (36). However, the *L. murinus* abundance is high in obese and atherosclerotic mice. Studies have found that antibiotic-induced ecological disorders, especially excessive growth of *L. murinus*, can impair intestinal metabolic functions and lead to the development of alopecia. Additionally, high salt intake has been linked to depletion of *L. murinus*, which has been associated with increased CD4^+^Rorγt^+^TH17 cells and blood pressure (31, 37, 38).

We next analyzed the most abundant members of Myoviridae, Siphoviridae, and Podoviridae in the microbial community structure of bacteriophages. Compared with those of Wt and ApoE^−/−^mice, the relative abundances of Podoviridae, Myoviridae, and Siphoviridae were significantly increased and decreased, respectively, in the intestinal tracts of ob/ob mice. Podoviridae has been associated with ulcerative colitis and type 2 diabetes and can aggravate these diseases (39). Myoviridae abundances are significantly increased (*p* < 0.05) in patients with type 2 diabetes, although the mechanism remains unclear (40, 41). Additionally, *Lactobacillus_ prophage_ Lj965* and *Lactobacillus _ prophage _ Lj771* abundances were significantly higher in ob/ob mice than in Wt mice. Studies have shown that *Lactobacillus* is significantly positively associated with Parkinson's disease (11, 42). The intestinal microbial composition is closely related to glucose homeostasis in the blood of obese mice (34), and a sugar-rich diet can induce *Lactobacillus* prophage lysis, which can profoundly impact the intestinal microbial community (43). Further study is needed to determine how gut microbes regulate glucose homeostasis in patients with metabolic diseases and provide microbial resources for developing new therapies for obesity and related metabolic disorders (44–46).

Herein, we have shown that the key microbial species in intestinal microorganisms of mice with metabolic diseases may play causal roles in the pathophysiology of metabolic diseases (47). To further support a potential causal relationship, the characteristics of intestinal metabolites must be analyzed to achieve similar metabolic disease characteristics and show a significant correlation with intestinal microorganisms. Additionally, in the ob/ob mouse model, the levels of several landmark metabolites, including short-chain fatty acids, DCA, LCA, GDCA, and glutamine, were altered. These microbial metabolites are related to the main intestinal microorganisms, i.e., *Lactobacillus, Bifidobacterium*, and *Enterobacter*, which promote lipid absorption, which affects triglycerides, cholesterol, glucose, and energy homeostasis (48, 49). *Lactobacillus* promotes the growth of pancreatic ductal carcinoma by activating tumor-associated macrophages through tryptophan in metabolic foods (37, 50). Our multi-omics analysis provides basic data to research the causal relationship between key microbial species and their metabolites in the occurrence and development of metabolic diseases, especially obesity.

Metabolite pathways analysis suggested that these metabolite pathways were mainly involved in aminoacyl-tRNA biosynthesis and are widely present in organisms. With the development of genome and exon sequencing technology and the discovery of new clinical cases, aminoacyl-tRNA synthetases (ARSs) are considered manifestations of metabolic diseases and are closely associated with obesity, atherosclerosis, and type 2 diabetes (3, 14, 51). The classic function of ARSs is to provide raw materials for protein biosynthesis (51). Increasing evidence suggests that ARSs play important roles in controlling inflammation, immune response (15, 48), tumorigenesis, and other important physiological and pathological processes. The availability of intracellular amino acids is closely related to the regulation of various cellular processes (44, 52). However, further work is needed to determine which gut bacteriophages, bacteria, and metabolites can be used as targets for metabolic diseases to develop nutritional interventions for obesity and atherosclerosis-related diseases.

## Data availability statement

The datasets presented in this study can be found in online repositories. The names of the repository/repositories and accession number(s) can be found in the article/Supplementary material. Ultra-deep metagenomic sequencing of the fecal samples was performed at Shanghai Biozeron Biotechnology Co., Ltd. The NCBI accession number for the metagenomic sequences reported herein is PRJNA755346.

## Ethics statement

The animal study was reviewed and approved by the animal experiments were carried out under NIH guidelines (NIH Publication No. 85-23 Rev. 1985) with supervision of Animal Experimentation Ethics Review Committee of South China Agricultural University (Guangzhou, China).

## Author contributions

XF and HW conceived and designed the research framework. SD performed the experiment, analyzed and interpreted the data, and prepared the original draft of the manuscript. CW revised the grammar of the manuscript and supplemented the discussion. YT and FZ analyzed the bioinformatics and constructed the bioinformatics graphs. JD assisted in the animal experiments. WH, RZ, and ZL revised and edited the manuscript. All authors contributed to the article and approved the submitted version.

## Funding

This work was supported by the National Natural Science Foundation of China (31871790, 31671855, and 81770434), the National Key Research and Development Program of China (2018YFC1313802), and the Key-Area Research and Development Program of Guangdong Province (2018B020205002).

## Conflict of interest

Authors YT and FZ were employed by the company Shanghai Biozeron Biotechnology Co., Ltd. The remaining authors declare that the research was conducted in the absence of any commercial or financial relationships that could be construed as a potential conflict of interest.

## Publisher's note

All claims expressed in this article are solely those of the authors and do not necessarily represent those of their affiliated organizations, or those of the publisher, the editors and the reviewers. Any product that may be evaluated in this article, or claim that may be made by its manufacturer, is not guaranteed or endorsed by the publisher.

## Supplementary material

Supplementary Figure S1 Body weight and blood glucose levels in the three mouse groups. **(A)** Changes in body weight in the t 31.68% of the changes, respectively hree mouse groups; **(B)** Changes in blood glucose in the three mouse groups. The same letter indicates no significant difference; different letters indicate significant differences.

Supplementary Figure S2 Proportions of gut archaea at the phylum, genus and species levels. **(A)** Proportions of gut archaea at the phylum level; **(B)** Proportions of gut archaea at the genus level; **(C)** Proportions of gut archaea at the species level.

Supplementary Figure S3 Proportions of gut bacteria at the phylum, genus and species levels. **(A)** Proportions of gut bacteria at the phylum level; **(B)** Proportions of gut bacteria at the genus level; **(C)** Proportions of gut bacteria at the species level.

Supplementary Figure S4 Proportions of gut bacteriophages at the family and species levels. **(A)** Proportions of gut bacteriophages at the family level; **(B)** Proportions of gut bacteriophages at the species level.

Supplementary Figure S5 Composition of intestinal metabolites in the three groups of mice.

Supplementary Figure S6 Bubble plot of the enriched KEGG pathways. Bubble size represents the number of metabolites enriched in the pathway.

Supplementary Figure S7 Differential expressions in the intestinal metabolic KEGG pathways in the three mouse groups. **(A)** Differential expressions in the synthesis and degradation of the ketone bodies pathways in the Wt and ApoE^−/−^ mice; **(B)** The KEGG metabolites pathway of aminoacyl tRNA biosynthesis was differentially regulated in the Wt and ob/ob mice; **(C)** Comparing the aminoacyl-tRNA biosynthesis metabolic pathways in ApoE^−/−^ and ob/ob mice. Red reflects upregulation; blue reflects downregulation.
