# Supplementary material for: Metagenomic and metabolomic analyses show correlations between intestinal microbiome diversity and microbiome metabolites in ob/ob and ApoE−/− mice
Source: Front Nutr. 2022 Oct 13;9:934294. doi: 10.3389/fnut.2022.934294 (PMC9634818; doi:10.3389/fnut.2022.934294)
Supplement: Supplementary file 1 [file Table_1.DOCX]

Supplementary table 1 The ingredients of dietary

| Ingredient | D12450J |
| --- | --- |
|  | gm% kcal% |
| **Casein, 80 Mesh** | 200 800 |
| L-Cystine | 3 12 |
| Corn Starch  Maltodextrin 10 | 506.2 2024.8  125 500 |
| Sucrose | 68.8 275.2 |
| Cellulose, BW200 | 50 0 |
| Soybean Oil | 25 225 |
| Lard | 20 180 |
| Mineral Mix S10026 | 10 0 |
| DiCalcium Phosphate | 13 0 |
| Calcium Carbonate | 5.5 0 |
| Potassium Citrate, 1 H2O | 16.5 0 |
| Vitamin Mix V10001 | 10 40 |
| Choline Bitartrate | 2 0 |
| FD&C Yellow Dye #5 | 0.04 0 |
| FD&C Red Dye #40 | 0 0 |
| FD&C Blue Dye #1 | 0.01 0 |
| Total | 1055.05 4057 |

Supplementary table 2 The internal standards

| Internal Standard | |
| --- | --- |
| C14 0_D3 | L_Tyrosine_D4 |
| C15 0_D3 | C13 0_D2 |
| D_Ribose_5C13 | L_valine_D8 |
| Citric acid_D4 | Ornithine_D6 |
| C2 0_D3 | L_Arginine_15N2 |
| C3 0_D3 | L_Lysine_D4 |
| Pyruvic acid_13C3 | L_Histidine_15N3 |
| aMCA_D5 | Glycine_D5 |
| UDCA_D4 | L_Alanine_D4 |
| Adipic acid_D8 | L_Serine_D3 |
| Suberic acid_D4 | L_Methionine_D3 |
| L_Lactic acid_D3 | L_Phenylalanine_D8 |
| 3_Hydroxybutyric acid_D4 | L_Aspartic acid_D3 |
| Succinic acid_D4 | L_Tryptophan_D5 |
| beta_Hydroxyisovaleric acid_D8 | Leucine_D3 |
| Mandelic acid_D5 | C4 0_D3 |
| 4_Hydroxyphenylacetic acid_D6 | TCDCA_D9 |
| Ethylmalonic acid_D3 | Carnitine_D3 |
| DL_Malic acid_D3 | L_Acetylcarnitine C2_D3 |
| Benzoic acid_D5 | L_velarylcarnitine C5_D3 |
| Hippuric acid_D5 | Octanoylcarnitine C8_D3 |
| Hydrocinnamic acid_D9 | L_Palmitoylcarnitine C16_D3 |
| Ketoleucine_D3 | CDCA_D4 |
| C5 0_D9 | C10 0_D2 |
| C6 0_D3 | C12 0_D3 |
| C8 0_D3 | GCA_D4 |
| C9 0_D3 | TCA_D4 |
| C11 0_D3 | GCDCA_D4 |
| CA_D4 | D_Glucose_D7 |
| LCA_D4 | D_Fructose_6C13 |
