# Supplementary figures and images for: Metagenomic and metabolomic analyses show correlations between intestinal microbiome diversity and microbiome metabolites in ob/ob and ApoE−/− mice
Source: Front Nutr. 2022 Oct 13;9:934294. doi: 10.3389/fnut.2022.934294 (PMC9634818; doi:10.3389/fnut.2022.934294)

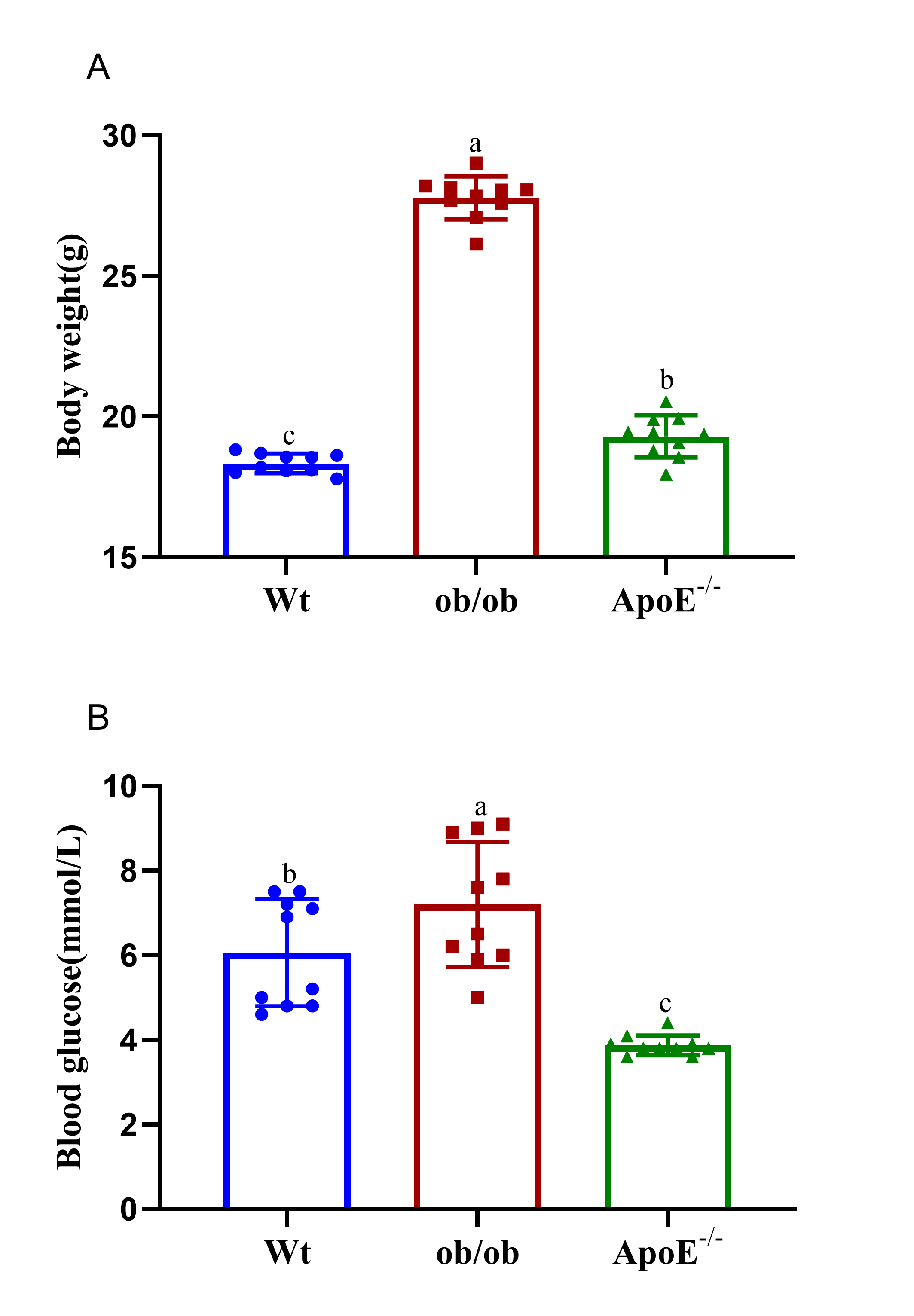

Supplement: Supplementary Figure S1 — Body weight and blood glucose levels in the three mouse groups. (A) Changes in body weight in the t 31.68% of the changes, respectively hree mouse groups; (B) Changes in blood glucose in the three mouse groups. The same letter indicates no significant difference; different letters indicate significant differences. [file Image_1.TIF]

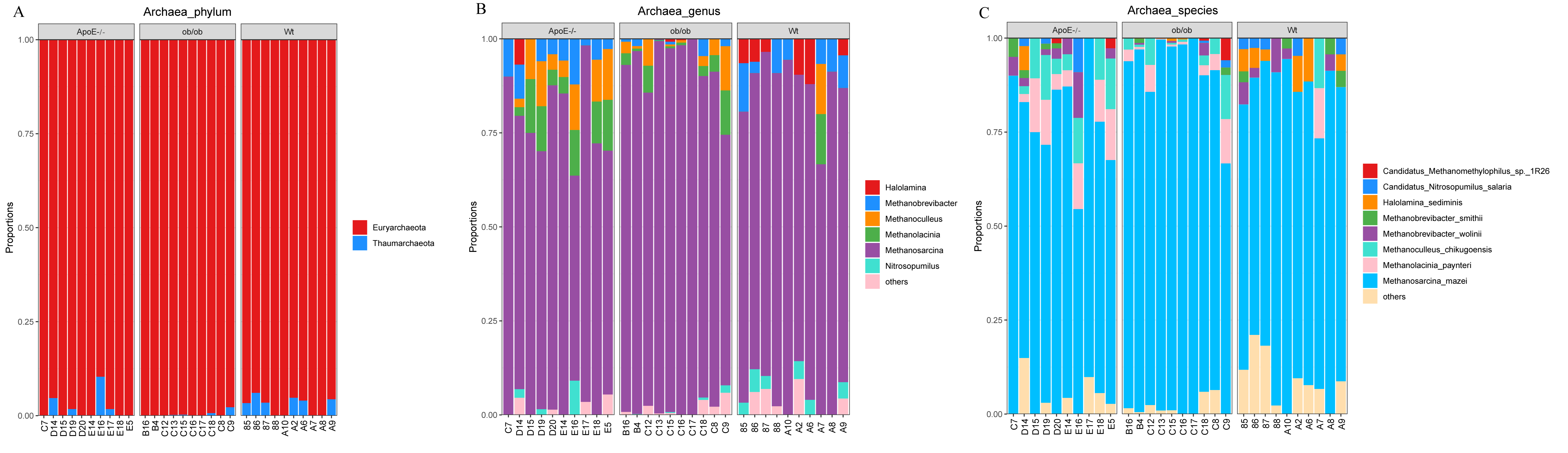

Supplement: Supplementary Figure S2 — Proportions of gut archaea at the phylum, genus and species levels. (A) Proportions of gut archaea at the phylum level; (B) Proportions of gut archaea at the genus level; (C) Proportions of gut archaea at the species level. [file Image_2.TIF]

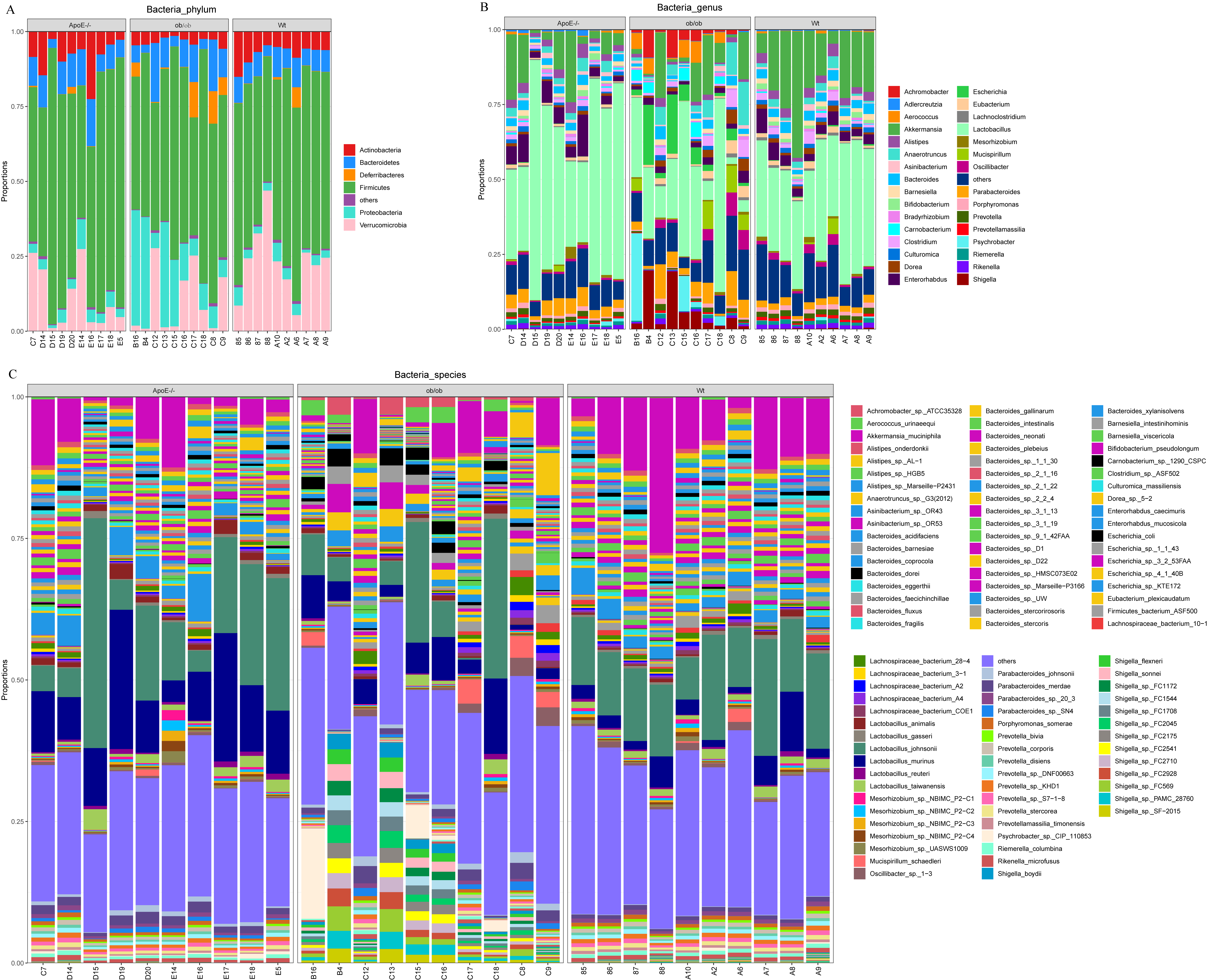

Supplement: Supplementary Figure S3 — Proportions of gut bacteria at the phylum, genus and species levels. (A) Proportions of gut bacteria at the phylum level; (B) Proportions of gut bacteria at the genus level; (C) Proportions of gut bacteria at the species level. [file Image_3.TIF]

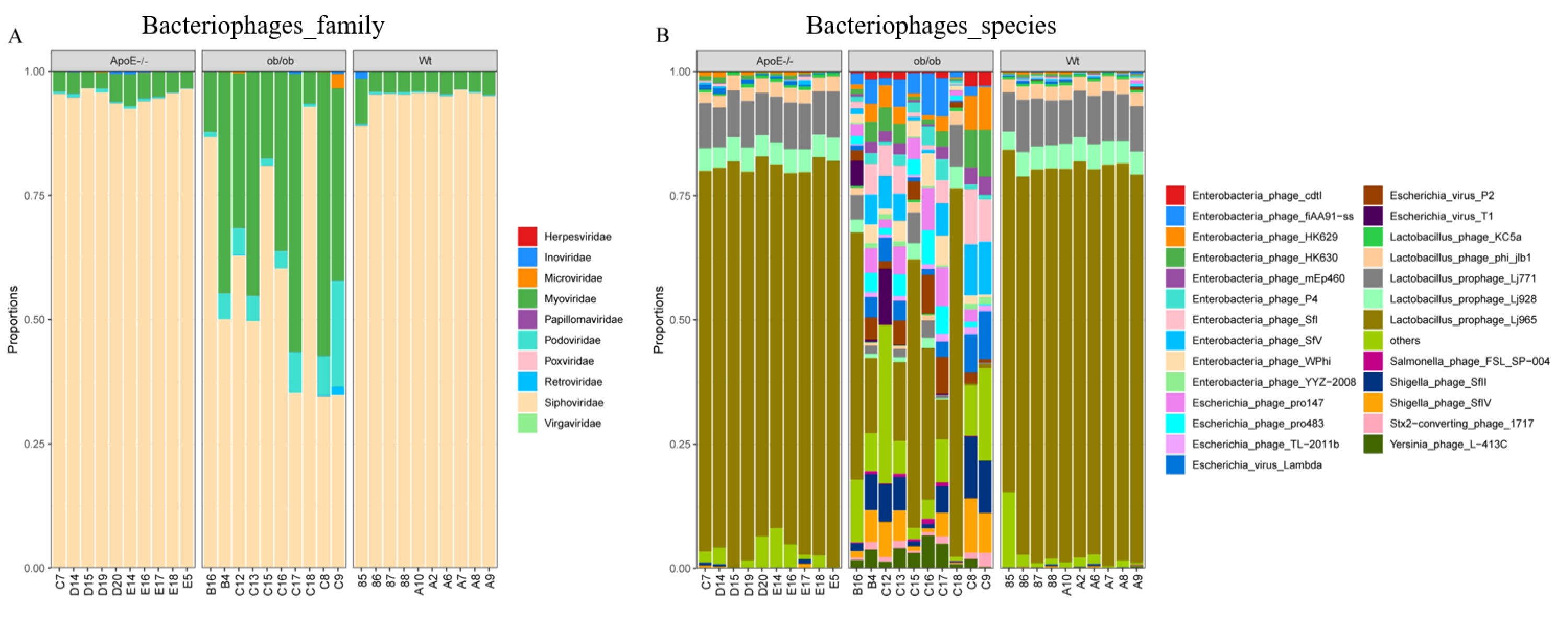

Supplement: Supplementary Figure S4 — Proportions of gut bacteriophages at the family and species levels. (A) Proportions of gut bacteriophages at the family level; (B) Proportions of gut bacteriophages at the species level. [file Image_4.TIF]

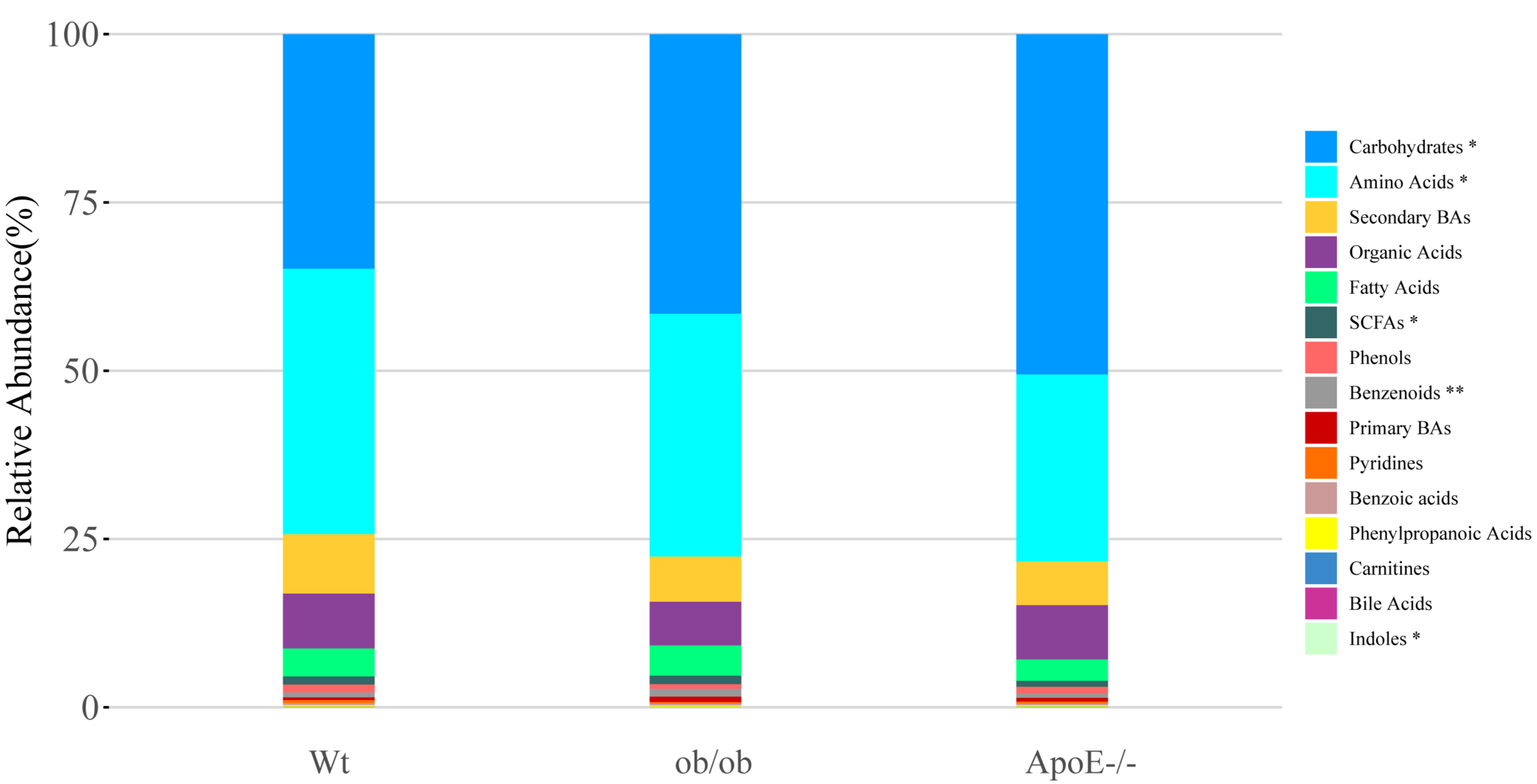

Supplement: Supplementary Figure S5 — Composition of intestinal metabolites in the three groups of mice. [file Image_5.TIF]

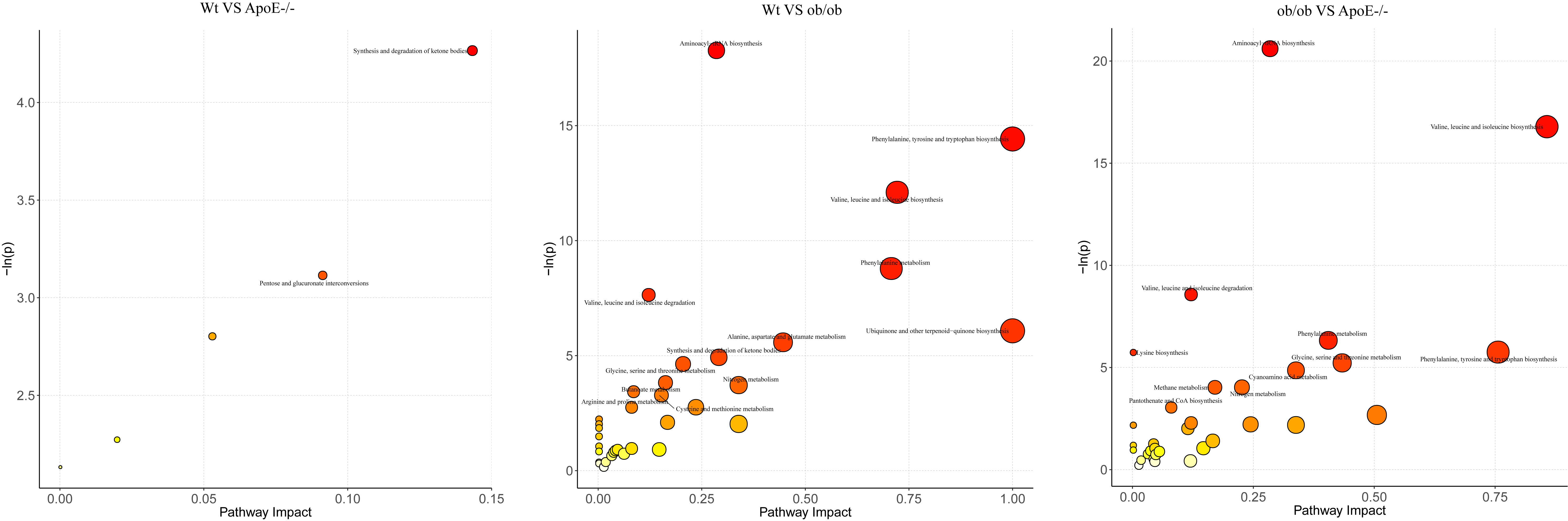

Supplement: Supplementary Figure S6 — Bubble plot of the enriched KEGG pathways. Bubble size represents the number of metabolites enriched in the pathway. [file Image_6.TIF]

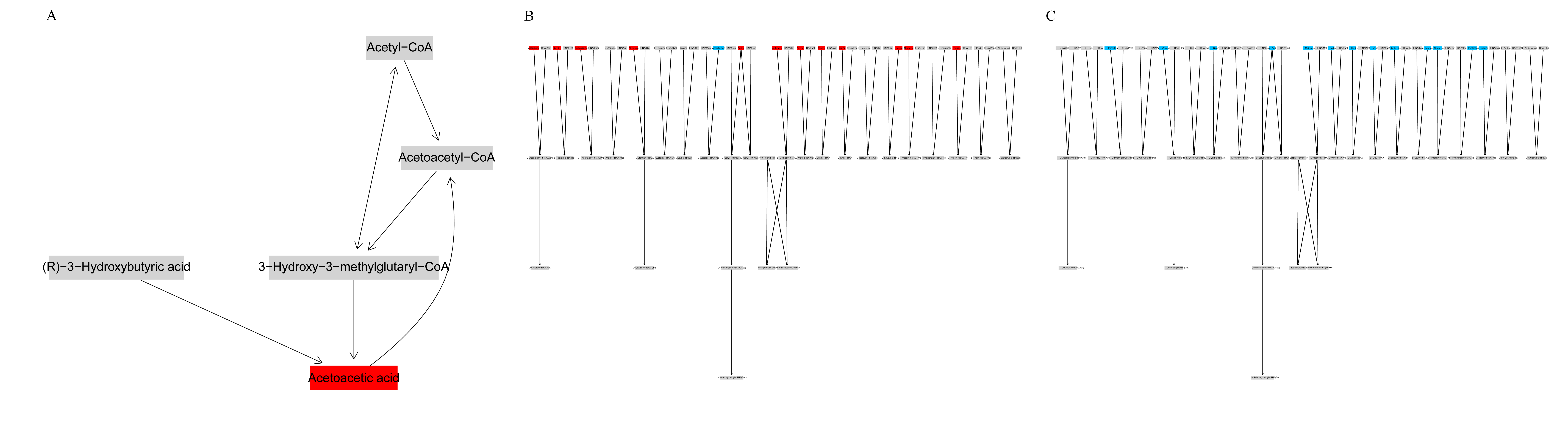

Supplement: Supplementary Figure S7 — Differential expressions in the intestinal metabolic KEGG pathways in the three mouse groups. (A) Differential expressions in the synthesis and degradation of the ketone bodies pathways in the Wt and ApoE−/− mice; (B) The KEGG metabolites pathway of aminoacyl tRNA biosynthesis was differentially regulated in the Wt and ob/ob mice; (C) Comparing the aminoacyl-tRNA biosynthesis metabolic pathways in ApoE−/− and ob/ob mice. Red reflects upregulation; blue reflects downregulation. [file Image_7.TIF]
